# Supplementary material for: Enhancing reactivity of SiO+ ions by controlled excitation to extreme rotational states
Source: Nat Commun. 2023 Jul 24;14:4446. doi: 10.1038/s41467-023-40135-x (PMC10366143; doi:10.1038/s41467-023-40135-x)
Supplement: Supplementary file 1 — Supplementary information [file 41467_2023_40135_MOESM1_ESM.pdf]

Supplementary Information

Enhancing reactivity of SiO^+ ions by controlled excitation to extreme rotational states

Sruthi Venkataramanababu[1,2], Anyang Li[3,*], Ivan Antonov[4], James Dragan[2], Patrick Stollenwerk[5], Hua Guo,[6] and Brian Odom[2,*]

[1]Applied Physics Program, Northwestern University, Evanston, 60201, IL, USA

[2]Department of Physics, Northwestern University, Evanston, 60201, IL, USA

[3]Key Laboratory of Synthetic and Natural Functional Molecule Chemistry, Ministry of Education, College of Chemistry and Materials Science, Northwest University, 710127 Xi'an, P. R. China

[4]Lebedev Physical Institute, Samara, 443011, Russian Federation

[5]Argonne National Laboratory, Lemont, 60439, IL, USA

[6]Department of Chemistry and Chemical Biology, University of New Mexico, Albuquerque, 87131, NM, USA

Supplementary Note 1: Trapping, optical pumping, and in-situ mass spectrometry

Experiments to measure the reaction rate are conducted in an ion trap where SiO^+ ions are co-loaded with Ba^+ ions. The SiO^+ ions are optically pumped into desired rotational states using a broadband optical pumping method. The techniques for ion trapping, in-situ mass spectrometry and optical pumping are described in Supplementary Figure 1.

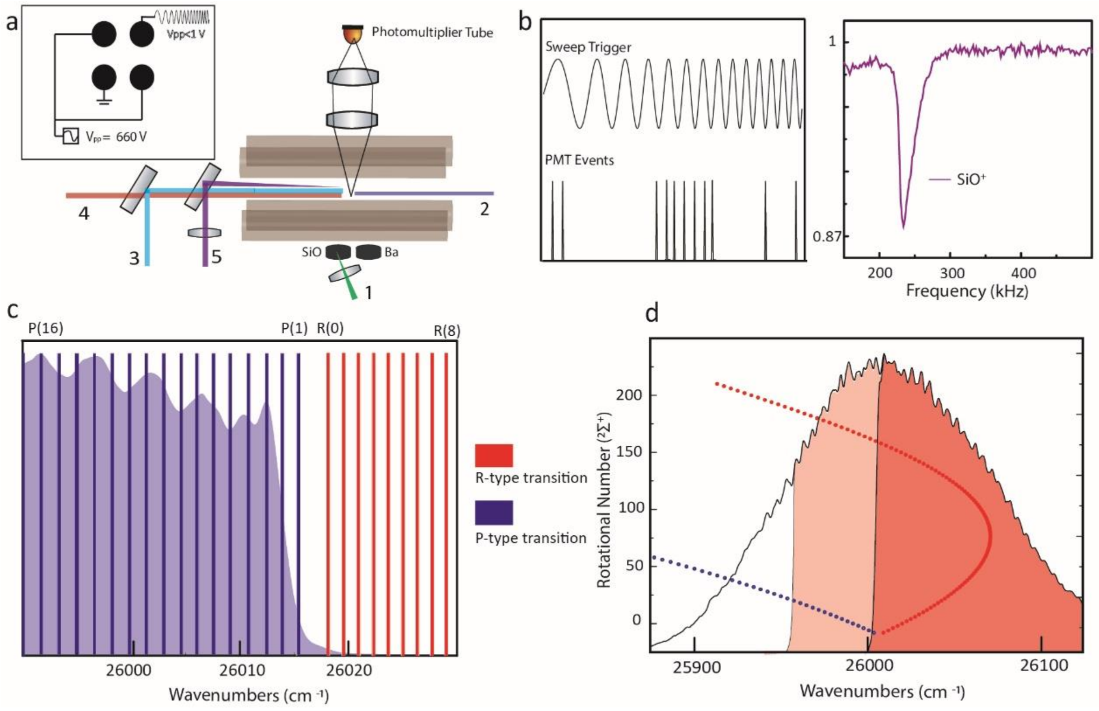

Supplementary Figure 1 a: The experimental setup consists of a linear ion trap, into which Ba^+ and SiO^+ ions are loaded via ablation (1) followed by photoionization (2). The Ba^+ ions are Doppler cooled continuously using the 493 nm (3) and 650 nm lasers (4). The Ba^+ cloud cools the translational motion of SiO^+. Rotational control is achieved via a broadband laser centered at 385 nm (5). Inset shows a top-view of the trap with the RF voltages and low-voltage chirp that is applied to the rods. **b: Left:** The waveform of the low-voltage chirp that is applied to the trap rods and the PMT events as a result of the low-voltage excitation. **Right:** When the chirp frequency resonates with the SiO^+ motion, the fluorescence of Ba^+ goes down; we monitor the center frequency of the dip in fluorescence and use that for in-situ mass spectrometry. **c:** Spectral mask used to pump SiO^+ to *j=1/2*. Blue (Red) sticks represent the P (R) branch, shaded region represents the intensity of light. To pump into *j=1/2*, all R branch transitions are blocked, allowing only P-type transitions. **d:** In step 1, (dark red), the spectral mask pumps SiO^+ to around *j =170* (super-rotors) by allowing only R-type transitions and completely blocking out all P -type transitions. In step 2 (light red), we expose an additional section of the spectra corresponding to R-type transitions to take the molecule to even higher rotational states (*j=170* and onwards).

Supplementary Note 2: SiO^+ population distribution calculations via Einstein rate equations

Spectra of the X-X, X-A, X-B and A-B systems were simulated with the PGOPHER package using ab initio calculations and experimental data from Rosner *et.al.*[1] Permanent electric dipole and transition dipole curves for the X, A, and B states were obtained from ab initio calculations.[2,3] De-perturbed vibrational constants were used in RKR inversion to reconstruct the potential energy curves of the X, A, and B states. The number of vibronic levels for RKR was limited to 3, i.e., *v* = 0 - 2 for all electronic states. Each vibrational level included rotational states *j* = 0.5 - 199.5. Optical pumping, radiative relaxation and interactions with blackbody radiation were then simulated by numerically solving a set of first-order rate equations for all 4800 states included in the simulation. Further details regarding the simulations can be found in the work by *J .Dragan et.al.*[4]

The population time evolution for t = 0 – 100 s is calculated starting from the initial state vector in which the population is equally distributed between *j* = 13.5 and *j*=15.5 of the *X,v*=0 level. The population evolution is largely complete at t = ~1s. Supplementary Figure 2a shows fractional populations in rotational levels of *X, v*=0 and *A, v=0* states at *t* =100 s. The selected vibronic levels account for 91.6% of total population with most of it being in the *A, v=0* state which becomes the ground vibronic state at high *j*. The remaining 8.4% of population is mostly located in the *A,v*=1 state. The mechanism of populating the high *j* levels in the *X* and *A* states is the following: pumping the highly diagonal *X-B* transition followed by radiative decay of the *B* state results in a quick increase of the rotational quantum number of the *X* state. Near *j* = 145.5, *A, v=0* state becomes lower in energy than *X, v*=0; however, the latter is sufficiently metastable to continue increase in *j* via the X-B transition pumping. The population of the A state slowly accumulates via the X-A and B-A radiative decay processes. The complex shape of population curves vs *j* in *X, v*=0 and *A, v*=0 is determined by perturbation between selected rotational levels of *X, v*=0 and *A, v*=0,1 states which occur near *j* = 140 and 165. The perturbation results in increased probability of radiative decay and pumping via the *B-A* transition due to intensity borrowing from *B-X*. The population in the excited rotational levels of the *A, v*=0 state slowly decays radiatively down the rotational level ladder until it reaches *j* = 145.5 at which point it may go back to *X, v*=0 state and undergo rotational pumping via *X-B*

transition by the laser field. Alternatively, it may undergo direct *A*-*B* excitation after which the molecule in the B state preferentially decays to *X* state and undergoes *X*-*B* excitation.

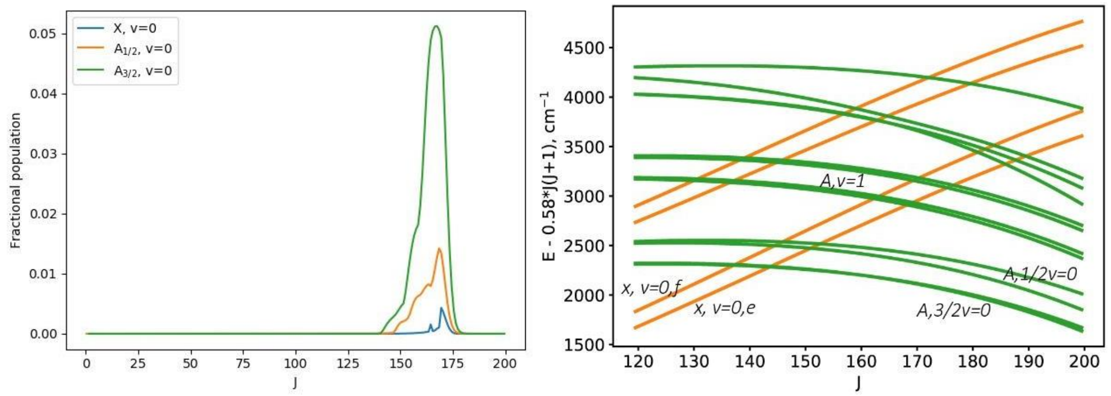

b

Supplementary Figure 2 a: Rotational populations of SiO^+ in *X,v*=0 and *A,v*=0 after 100 seconds of optical pumping. **b:** Energies of *X*, *v* and *A*, *v* states as a function of *j* at *j* > 120. The quantity 0.58**j(j+1)* has been subtracted to flatten out the curves. The orange lines represent *X, v*=0 - 1*, F1e and F2f manifolds.* The green lines represent the A, v = 0 - 2 states, F1e, F1f, F2e and F2f manifolds.

The *X, v*=0 state curves for e and f parities are the true ground state at *j* = 120, but they cross *A, v* = 0 curves near *j* = 140 - 150 and *A, v* = 1 curves near *j* = 165 – 175 as shown in Supplementary Figure 2b. Near the curve crossings the electronic states perturb each other through rotational Hamiltonian and the *A*-*B* transition which is normally very weak borrows intensity from the *X*-*B* transition. This effectively means that the *A*, *v*=0 levels near *j* = 140-150 can be pumped via *A, 0* - *B, 0* transition, and *A, v* =1 levels near *j* = 165-175 are pumped via the *A, 1* - *B, 0* transition. However, outside of these *j* regions *A, 0* and *A, 1* rotational states are essentially "dark" and therefore relax radiatively to lower *j* values until they either reach the perturbed region or the point where *X*, *v* = 0 is the true ground state. In the perturbed regions, *A* - *B* excitation has a high probability of the upper state decay to the *X, v = 0* which will result in subsequent *X* - *B* pumping. Therefore, the overall process results in rotational heating of SiO^+ molecules, populating the *A* state and maintaining the super-rotors rotational population through *X*-*B* pumping.

Supplementary Note 3: Gaussian Decomposition

The LCFMS mass spectrum has a m/Δm of ~ 30 and thus it cannot resolve the SiO^+ and $SiOH^+$ peaks. However, the mass spectrum can be fitted to a sum of two Gaussians centered at two different frequencies corresponding to the secular frequencies of SiO^+ and $SiOH^+$. As the SiO^+ reacts to form $SiOH^+$, the amplitude of the SiO^+ Gaussian drops while that of the $SiOH^+$ Gaussian rises. Consequently, the effective Gaussian shifts towards lower frequencies as the reaction proceeds indicating an increase in the $SiOH^+$ concentration in the trap.

Mathematically, by writing a Gaussian function in a Taylor's series expansion, it is possible to show that,

$$G(x-x_{0,3}) = a*G(x-x_{0,1}) +(1-a) *G(x-x_{0,2}), \qquad \text{(i)}$$

where $G(x-x_{0,i})$ is a Gaussian centered around $x_{0,i}$. The two Gaussians centered around $x_{0,1}$ and $x_{0,2}$ are assumed to have the same width. Here, $x_{0,3} =(x_{0,1}-x_{0,2}) *a+x_{0,2}$, here $x_{0,3}$ $x_{0,2}$ and $x_{0,1}$ are the centers of the effective Gaussian, Gaussian centered at SiO^+ and Gaussian centered at $SiOH^+$ respectively, and '*a*' is the amplitude of the Gaussian centered at SiO^+.

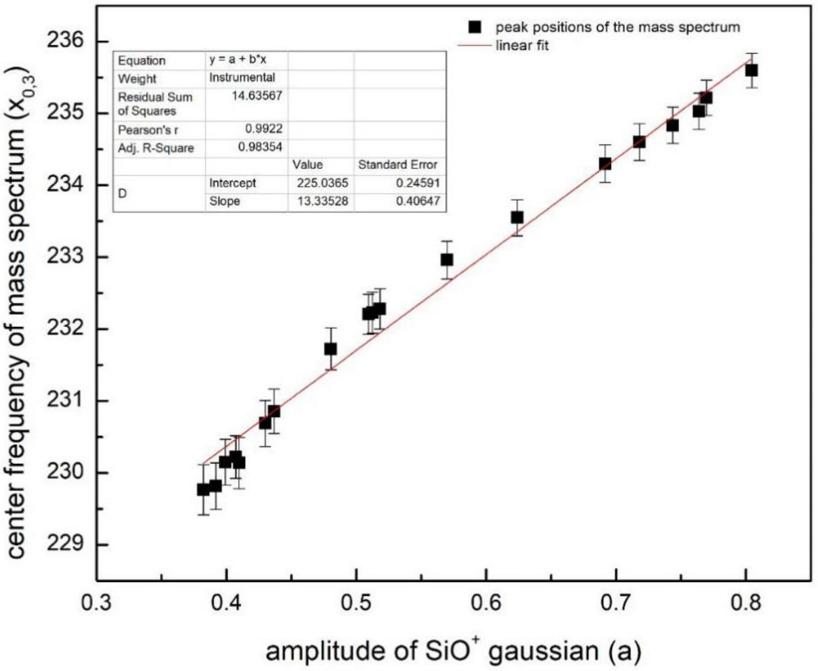

Supplementary Figure 3 Linearity between the amplitude of SiO^+ and the position of the peak of the mass spectrum.

Supplementary Note 4: Singular value decomposition

As SiO^+ reacts away to form $SiOH^+$, the dip in fluorescence shifts to lower frequencies, reflecting an increase in $SiOH^+$ concentration. The resolving power of the LCFMS technique in our ion trap is m/Δm= 30 and thus it cannot resolve between SiO^+ (mass 44) and $SiOH^+$ (mass 45). Moreover, the fluorescence of Ba^+ fluctuates due to drifts in frequency, on the order of a few MHz, of the lasers used to Doppler-cool Ba^+. On rare occasions, this also led to a loss of SiO^+ ions from the trap if the drift was larger than 5 MHz. To deal with the noise from fluorescence fluctuations, we used singular value decomposition (SVD) of the data to effectively isolate amplitude variations in fluorescence and mass-dependent frequency shift of the fluorescence spectrum. With sufficient averaging, and by appropriately employing SVD analysis, we inferred the rate of reaction of SiO^+.

The 2-D matrix (***R***) in Supplementary Figure 4a is the experimental data; it represents the depletion in Ba^+ fluorescence as function of the chirp frequency and time. R has dimensions ***m x n***, ***m*** for chirp frequency and ***n*** for time. SVD factorizes ***R*** such that $\boldsymbol{R = U \Sigma V^*}$ where ***U*** has the dimensions of ***m x m*** and ***V*** has dimensions of ***n x n*** and ***U*** and ***V*** are unitary matrices. $\boldsymbol{\Sigma}$ is a diagonal ***m x n*** matrix with the diagonal entries being ordered from the largest to smallest values. SVD of any matrix can be used to obtain a weighted and ordered sum of several constituent matrices. We now rewrite ***R*** as $\boldsymbol{R} = \boldsymbol{\Sigma_i \sigma_i U_i V_i}$, where $\boldsymbol{\sigma_i}$ are the ordered values from the diagonal of $\boldsymbol{\Sigma}$ and $\boldsymbol{U_i}$ and $\boldsymbol{V_i}$ are ith columns of ***U*** and ***V**** respectively. $\boldsymbol{\sigma_i}$′ s weigh the contribution of each of the separable matrices $\boldsymbol{U_i V_i}$ to the original matrix. Writing the original matrix in terms of its SVD decomposition reveals characteristic patterns in data that may otherwise not be noticeable due to noise and size of the data. In our case, we would like to understand the kinetics of $SiOH^+$ production and the consequent shift in the fluorescence spectrum to lower frequencies by isolating it from the intensity and linewidth fluctuations in fluorescence. The first few values of $\boldsymbol{\sigma_i}$ are of appreciable intensity, the rest are very small positive numbers indicating that the first few elements of $\boldsymbol{\Sigma_i \sigma_i U_i V_i}$ contribute to the actual data and the rest can be discarded without affecting the information in the data. In our case, we choose to work with the first two singular values. Supplementary Figure 4b represents the first two columns of ***V***, the blue trace is $\boldsymbol{\sigma_1 V_1}$ and shows the fluorescence depletion spectrum as a function of frequency. The dotted lines show $\boldsymbol{\sigma_2 V_2}$ which represents the change in the shape of the spectrum - the spectrum shifts to lower frequencies. The orange line is the time

dependence of the frequency shift in the spectrum and that is given by by plotting $\boldsymbol{\sigma_2 U_2}$. We fit this using an exponential function of the form $\boldsymbol{y = y_0 e^{-kt}}$. The decay constant ($k$) gives the rate of formation of $SiOH^+$.

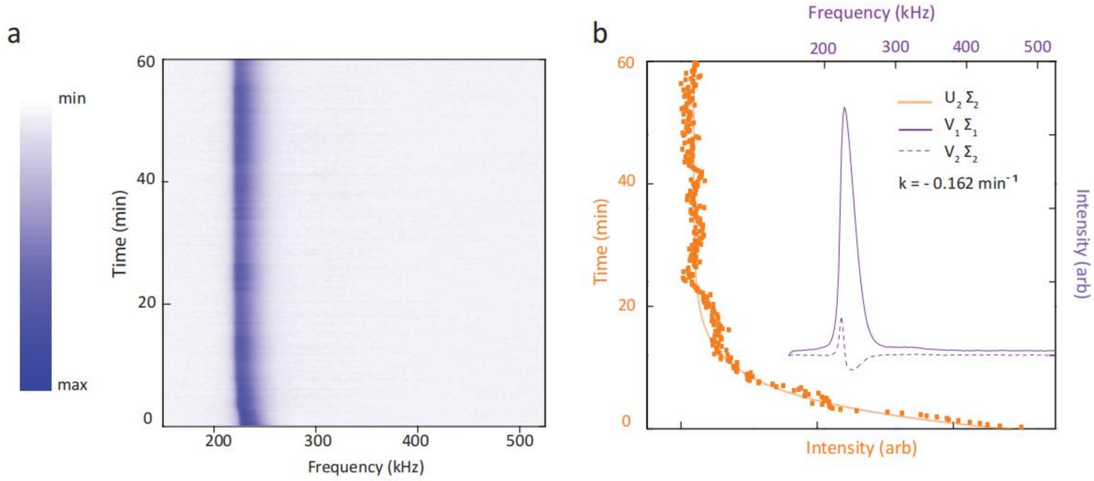

Supplementary Figure 4 **a**: Experimental data showing the fluorescence of Ba^+ as a function of the RF chirp frequency and time. **b**: Selected components from the singular value decomposition of the data set shown in ***a***.

SVD can separate out several components of the data such as the mass spectrum, change in the mass spectrum with time and the broadening of the mass spectrum (described by $\boldsymbol{U_1\ \sigma_1}$, $\boldsymbol{U_2\ \sigma_2}$, and $\boldsymbol{U_3\ \sigma_3}$ respectively; these vectors are also orthogonal to each other). We then fit only the change in the peak as a function of time to an exponential decay, (neglecting amplitude fluctuations in the fluorescence and broadening effects). Since we already know that there is a linear dependency between the change in the center of the peak and the fraction of SiO^+ in the system, the fit for the change in the center of the peak also gives us the rate of the reaction.

Supplementary Note 5: Validation of the fitting analysis

Given the complexity of the fitting analysis described in Section III, we validate the method using a simpler, albeit significantly more time-consuming, approach. To ensure that there were no unknown systematic effects from the method of using secular frequencies for rate extraction, a direct approach for measuring the fraction of species near mass 44 able to be

dissociated was performed. In this method, SiO^+ molecules were loaded into the trap. After a specified time, all the unreacted molecules were dissociated using the X -> C transition.[2,3] Any remaining molecules that showed up as a dip in the secular frequency, therefore, corresponded to $SiOH^+$. The fraction dissociated was used to determine the fraction of SiO^+ reacted. The fraction of reacted SiO^+ was plotted as a function of time and fitted to an exponential decay function with only the decay constant as a free parameter. The fit resulted in a decay constant of 16(3) min at a H_2 density of $11*10^6$ cm^{-3}. This is consistent with our observed reaction rates at the same pressure, thereby validating the secular frequency approach used in our measurements.

Despite the more straightforward appeal of these measurements, they take an exceedingly long time to accomplish and therefore, the secular frequency measurements were preferred over these measurements once validated.

Supplementary Note 6: Measurement of the reaction rate constant and uncertainty in the measurement

The bi-molecular rate coefficients were obtained by measuring the reaction rates as a function of H_2 concentration. In Supplementary Figure 5, the measured reaction rate is shown as a function of estimated H_2 concentration in the trap. The lowest hydrogen concentrations were performed at the baseline pressure of the vacuum system which slowly decreased over several months of pumping with an ion pump and a titanium sublimation pump (TSP). For these measurements, we use the nascent post-photo-ionization rotational distribution of SiO^+ with population in states j= 13 - 16; the rate of reaction for the un-pumped nascent distribution is indistinguishable from the thermal distribution at 300K and was verified experimentally to be the case. To estimate H_2 concentration in the trap, we assume that ~75% of the background gas is H_2. The ion gauge reading is corrected using appropriate gas correction factors. In Supplementary Figure 4, uncertainties for data points at low H_2 densities ($7x10^6$ cm^{-3}, $14x10^6$ cm^{-3}), are obtained from standard deviations in the sample data; for higher number densities, only one data set was available to measure the estimated reaction

rate. The error bars for these have been conservatively estimated at 30% based on the data sets taken at lower H_2 pressure.

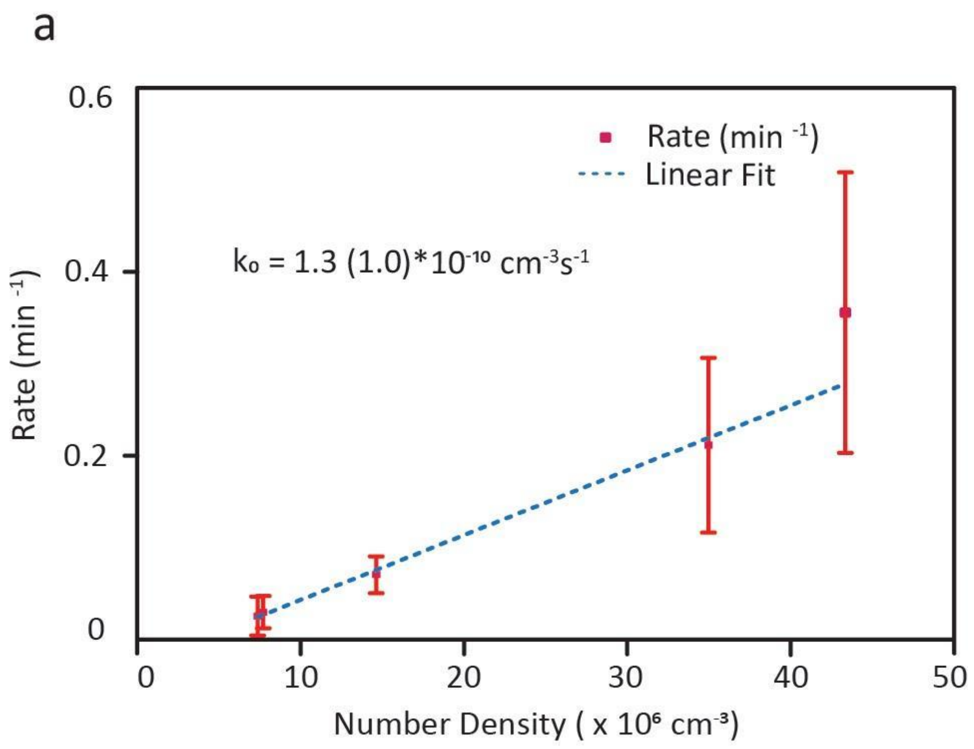

Supplementary Figure 5: Experimental data showing the fluorescence of Ba^+ as a function of the RF chirp frequency and time Stern-Volmer plot depicting the reaction rate as a function of H_2 number density; slope obtained from the linear fit gives us the bi-molecular rate constant for the reaction.

We repeated the measurement of the reaction rate at a given pressure several times over the course of a 5-day measurement period and during this 5-day period, the pressure reading was constant to within the precision of the gauge.

The slope of the straight-line extracted from the linear fit gives us the rate of the bi-molecular reaction: 1.3 (1.0) * 10^{-10} $cm^{-3}s^{-1}$. The previously reported rate constant measurement by Fahey et al. (3.2 (1.0) * 10^{-10} $cm^{-3}s^{-1}$) lies within 2σ of our measurement. The large discrepancy between the two measurements may be due to the uncertainty of the

gas composition and the ion gauge reading in our measurements. We must emphasize that this uncertainty, however, has no bearing on the observed reaction rate enhancement as all the rates for the enhancement measurement were measured at a fixed pressure corresponding to an estimated hydrogen density of 7×10^6 cm^{-3}. By performing the comparison at a fixed pressure, all plausible systematic effects that could contribute to the observed enhancement with rotations are common mode. Thus, the large uncertainty of the estimated rate coefficient and any source for the discrepancy with Fahey et al do not contribute to the uncertainty of the relative enhancement. Therefore, the only relevant uncertainty for the observation of the enhancement is the statistical uncertainty shown in the plot in Fig. 2a.

As an aside, the Stern-Volmer plot is also an important test of linearity and establishes confidence in the fact that the gas composition does not change drastically at different pressures.

Our confidence in the gas composition being dominated by H_2 is further boosted by null results in reaction with Ba^+, Si^+, $SiOH^+$ (all of which are present in our trap) with several hundreds of hours of data taking spread over a few years.

Supplementary Note 7: Potential Energy Surface

Similar to our previous work on ion-molecule reactions,[5,6] the global potential energy surface (PES) of $SiOH_2^+(X^2A)$ consists of an analytical long-range (LR) interaction term and fitted PES from *ab initio* points in the short range. The LR term in the $SiO^+ + H_2$ reactant channel asymptote, which is important for an accurate description of the reaction kinetics at low temperatures, includes the leading electrostatic interactions between the two reactants when they are far apart. On the other hand, the fitted PES describes all other regions where chemical interaction dominates. These two segments are connected smoothly via a switching function, as discussed below.

a. Long-range part of the PES

In describing the LR interaction, the ion SiO^+ is approximated as a point charge, and q denotes the charge of the molecular ion (+1). H_2 is a homonuclear diatomic molecule without a dipole moment. Its parallel and perpendicular components of the polarizability are $\alpha_{||}$=0.4793 Å^3 and $\alpha_{\perp}$=0.1653 Å^3, and its overall polarizability can be approximated as $\alpha =$

$\alpha_{||} + 2\alpha_{\perp}$=0.81 Å^3. It also has a quadrupole moment (Θ=0.63 esu).[7] The long-range interaction between the ion SiO^+ and molecule H_2 is dominated by the leading electrostatic terms, which is proportional to the inverse powers of the distance between the two moieties. Specifically, the electrostatic potential V_{ES} includes charge-quadrupole, $V_{q-\Theta}(\xi, \theta)$, and charge-induced dipole, $V_{q-\alpha}(\xi, \theta)$, interactions:[8]

$$V_{ES} = V_{q-\Theta} + V_{q-\alpha} = \frac{1}{2}q\Theta\xi^{-3}(3\theta - 1) - \frac{1}{2}q^2\xi^{-4}[\alpha + \frac{\alpha_{||}+\alpha_{\perp}}{3}(3\theta - 1)] \quad \text{(ii)}$$

where ξ is the separation between the center of charge of the SiO^+ ion and the center of mass of the hydrogen molecule, and θ is the angle between ξ and the H-H internuclear bond vector. The center of charge is defined as $\vec{c}_{SiO^+} = (\sum_i q_i\vec{c}_i)/(\sum_i q_i)$ in analogy to the center of mass, where $\vec{c}_i$ are the atomic coordinates and the atomic charges q_i were calculated using the electrostatic potential on a grid (CHelpG) point selection algorithm.[9]

The LR PES can be written as:

$$V_{LR} = V_{SiO^+} + V_{H_2} + V_{ES} \quad \text{(iii)}$$

where V_{SiO^+} and V_{H_2} are the potential energy curves (PECs) for the isolated SiO^+ and H_2 molecules, which were spline-interpolated from *ab initio* data points at the level of UCCSD(T)-F12b (explicitly correlated unrestricted coupled cluster singles, doubles and perturbative triples)[10] with the correlation-consistent polarized core-valence quadruple-zeta basis set for explicitly correlated wavefunctions (cc-pCVQZ-F12).[11] The explicit treatment of the electron-electron interaction (F12) allows a fast convergence with respect to the complete basis set limit.[12] All the potential energies are related to the potential minimum with the SiO^+-H_2 separation of 100.0 Å.

It should be noted that the adiabatic potential V_{SiO^+} is the lower adiabat of the $X^2\Sigma^+$ and $A^2\Pi$ states of SiO^+ (as shown in Supplementary Figure 6). Since the SiO^+ species in the SiO^+ + H_2 → $SiOH^+$ + H reaction is always in the lowest vibrational level under experimental conditions, it is safe to assume that the reaction is well described on the adiabatic ground state PES of $SiOH_2^+$ in the reactant asymptote. However, it is conceivable that the A state might be involved in the strongly interacting region, particularly when SiO^+ is in a super rotor state. The neglect of non-adiabatic effects here is one of the possible reasons for the quantitative discrepancy of the calculated results from the experiment at high rotational states.

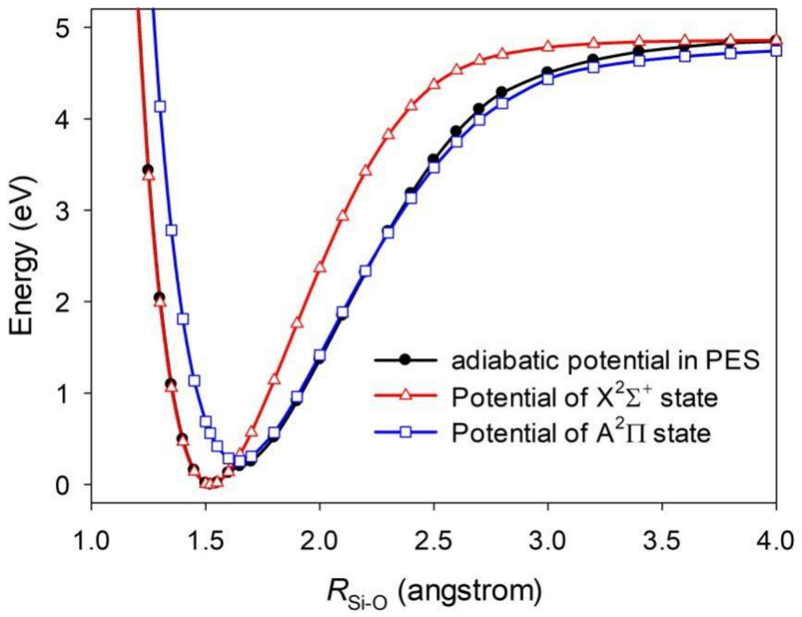

Supplementary Figure 6: The potential energy curves of SiO^+. The potentials of X and A state are calculated at the MRCI/cc-pCVQZ-F12 level.

b. Fitted PES

For the short-range PES, *ab initio* calculations were carried out at the level of UCCSD(T)-F12b/cc-pCVQZ-F12, the same as the PEC calculations of SiO^+ and H_2. All *ab initio* calculations were performed with MOLPRO.[13] The stationary points along the reaction path, along with their geometries, relative energies and harmonic frequencies are listed in Supplementary Tables 1 and 2.

To generate the *ab initio* points for fitting the PES, grids of points in the appropriate coordinates along the reaction pathway were first calculated in various regions. In total, 20147 points were fit using the high-fidelity permutation invariant polynomial-neural network (PIP-NN) method.[14] In particular, 17 PIPs up to second order have included as the input layer of the NN in order to take advantage of the permutation symmetry of this ABC_2 system. Morse-like variables, $p_{ij} = \exp(-r_{ij}/a)$, were used to construct the PIPs with a = 2.0 Å as the length parameter, in which r_{ij} denotes the internuclear distance between the ith and j^{th} atoms. The NN consists of 2 hidden layers, each with 30 and 60 interconnected neurons. The total number of parameters is thus 2460. In each NN fitting, the data were divided randomly into three sets, namely the training (90% of the data points), validation (5%), and test (5%)

sets. The early stop method was used to avoid overfitting.[15] The final PIP-NN PES was chosen as the average of three best fits, as suggested by the NN ensemble approach to minimize random errors.[16] The RMSEs for the training/validation/test sets of the three best fits are 16.5/16.9/20.4, 17.3/18.2/21.2, and 17.7/18.2/21.2 meV, respectively. The overall RMSE of the PES is 16.4 meV. The geometries and energies of the stationary points on the PES is compared in Supplementary Tables 1 and 2. The agreement with the values determined directly from *ab initio* calculations is quite satisfactory.

The overall PES is switched smoothly from the long-range PES to the fitted PES:

$$V = SV_{fit} + (1-S)V_{LR} \qquad \text{(iv)}$$

where the switching function is defined:

$$S = \frac{1-\tanh(3(R-7.0))}{2} \qquad \text{(v)}$$

where R is the distance between the center of mass of the two reactants. As a result, the overall PES is dominated by V_{LR} for $R > 7.5$ Å, while by V_{fit} $R < 6.5$ Å.

Supplementary Table 1: Energies and geometries in internal coordinates of the stationary points (IM and TS stand for intermediate and transition state, respectively) on the PES for the $SiO^+ + H_2 \rightarrow SiOH^+ + H$ / $Si^+ + H_2O$ reaction.

Species		Δ*E* (eV)	r_{SiO} (Å)	r_{HH} (Å)	r_{OH} /r_{SiH} (Å)	ϑ_{SiOH} /ϑ_{OSiH} (°)	ϕ_{SiOHH} (°)
$SiO^+ + H_2$	*ab initio*	0.0	1.63	0.74			
	PES	0.0	1.62	0.74			
$SiOH^+ + H$	*ab initio*	-1.76	1.54		0.96/2.50	180.0/0.0	
	PES	-1.76	1.54		0.96/2.50	180.0/0.0	
$Si^+ + H_2O$	*ab initio*	-0.50		1.52	0.96/-		
	PES	-0.50		1.51	0.96/-		
IM1 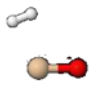	*ab initio*	-0.33	1.53	0.77	2.87/2.11	44.3/115.9	0.0
	PES	-0.31	1.51	0.76	2.81/2.10	49.9/118.5	0.0
IM2 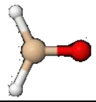	*ab initio*	-1.11	1.61	2.66	1.46/2.58	31.2/114.3	0.0
	PES	-1.12	1.61	2.66	1.47 /2.59	30.8/115.1	0.0
IM3 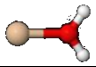	*ab initio*	-2.61	1.87	1.59	0.97/-	124.8/18.2	180.0
	PES	-2.60	1.84	1.60	0.97/-	123.3/17.9	180.0
IM4	*ab initio*	-3.01	1.57	3.48	0.96/1.49	133.2/111.9	0.0
	PES	-3.02	1.58	3.48	0.97/1.50	133.1/111.9	0.0

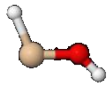							
IM5 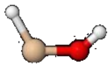	*ab initio*<br>PES	-2.92<br>-2.92	1.56<br>1.58	3.10<br>3.16	0.96/1.50<br>0.97/1.51	138.3/120.2<br>135.3/120.8	180.0<br>180.0
TS0* 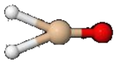	*ab initio*<br>PES	—<br>1.58	—<br>1.53	—<br>1.33	—<br>-/1.74	—<br>11.9/157.6	—<br>0.0
TS1 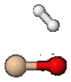	*ab initio*<br>PES	-0.11<br>-0.12	1.61<br>1.61	0.81<br>0.81	1.60/2.37<br>1.60/2.36	84.7/76.2<br>84.8/67.4	0.0<br>0.0
TS2 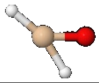	*ab initio*<br>PES	-0.14<br>-0.15	1.56<br>1.56	3.01<br>3.01	1.54/1.57<br>1.53/1.57	61.0/58.5<br>60.9/58.6	0.0<br>0.0
TS3 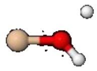	*ab initio*<br>PES	-0.69<br>-0.68	1.63<br>1.63	1.84<br>1.84	1.46/2.39<br>1.45/2.38	131.6/22.8<br>132.5/22.3	180.0<br>180.0
TS4 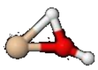	*ab initio*<br>PES	-1.07<br>-1.04	1.69<br>1.68	2.08<br>2.11	1.27/1.74<br>1.24/1.72	70.1/43.5<br>70.1/42.8	180.0<br>180.0
TS5 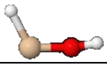	*ab initio*<br>PES	-2.89<br>-2.69	1.54<br>1.53	3.39<br>3.42	0.96/1.51<br>0.96/1.51	170.5/117.5<br>172.3/118.6	180.0<br>180.0

* TS0 is not optimized by UCCSD(T)-F12 method.

Supplementary Table 2. Harmonic frequencies of the stationary points on the PES for the $SiO^+ + H_2 \rightarrow SiOH^+ + H$ /$Si^+ + H_2O$ reaction

Species		Frequency (cm^{-1})					
		1	2	3	4	5	6
$SiO^+ + H_2$	*ab initio*	1150.7	4403.7				
	PES	1145.8	4401.0				
$SiOH^+ + H$	*ab initio*	354.1	1145.3	3846.1			
	PES	353.4	1143.8	3842.6			
$Si^+ + H_2O$	*ab initio*	1649.5	3835.0	3945.6			
	PES	1649.4	3831.9	3942.1			
IM1	*ab initio*	217.6	304.4	535.5	747.9	989.7	3970.4
	PES	246.7	263.5	490.2	714.2	1196.5	4006.1
IM2	*ab initio*	506.1	683.8	841.2	1002.2	2282.5	2357.1
	PES	499.1	676.7	838.3	985.8	2287.7	2365.8
IM3	*ab initio*	402.1	487.9	665.8	1654.3	3678.4	3770.9
	PES	465.0	496.0	661.2	1655.0	3551.2	3794.7
IM4	*ab initio*	540.4	593.9	720.0	1079.0	2084.4	3812.8
	PES	539.0	587.4	720.1	1083.3	2100.8	3839.7
IM5	*ab initio*	441.3	447.7	696.9	1113.8	2037.7	3812.2
	PES	440.6	450.6	695.7	1112.3	2034.6	3808.5

TS0	*ab initio*	-	-	-	-	-	-
	PES	266.3	649.7	1440.3	1820.2	335.9i	1828.0i
TS1	*ab initio*	350.7	405.8	701.6	939.1	3212.8	464.6i
	PES	350.9	418.2	699.4	964.4	3117.9	476.4i
TS2	*ab initio*	454.1	587.2	1072.4	1945.0	2219.3	1437.1i
	PES	473.1	605.7	1067.6	1959.6	2201.7	1428.2i
TS3	*ab initio*	359.0	596.8	908.8	1077.2	3747.8	2845.7i
	PES	408.7	593.7	906.8	1076.1	3744.9	2831.8i
TS4	*ab initio*	699.9	880.1	1693.0	3610.9	119.8i	1712.3i
	PES	826.1	928.0	1944.0	3617.9	323.8i	1764.6i
TS5	*ab initio*	268.8	689.4	1154.1	1997.8	3885.0	373.7i
	PES	269.9	687.7	1152.6	1993.7	3881.7	374.1i

Supplementary Note 8: Reaction Rates from Figure 2a.

	**Reaction rates (min^{-1})**
**No optical pumping**	0.09
	0.035
	0.058
	0.081
	0.055
	0.058
	0.032
	0.033
	0.040
	0.034
	0.045
	0.061
	0.080
	0.059
	0.066

	Reaction rates (min $^{-1}$)
Pump to j~57	0.091
	0.126
	0.100
	0.101
	0.080
	0.080
	0.072
	0.120
	0.151

	Reaction rates (min $^{-1}$)
Pump to super-rotor states	0.142
	0.147
	0.201
	0.182
	0.182
	0.222
	0.193
	0.163
	0.120
	0.151
	0.226
	0.184
	0.221

References

1 Rosner, S. D., Cameron, R., Scholl, T. J. & Holt, R. A. A Study of the $X^2\Sigma^+$ and $A^2\Pi$ States of SiO^+ Using Fast-Ion-Beam Laser Spectroscopy. *J. Mol. Spectrosc.* **189**, 83-94, doi:10.1006/jmsp.1997.7522 (1998).

2 Antonov, I. O. *et al.* Precisely spun super rotors. *Nat. Commun.* **12**, 2201, doi:10.1038/s41467-021-22342-6 (2021).

3 Qin, Z., Bai, T., Zhao, J. & Liu, L. Transition Properties between Low-lying Electronic States of SiO^+. *J. Mol. Spectrosc.* **370**, 111298, doi:10.1016/j.jms.2020.111298 (2020).

4 Dragan, J. B., Antonov, I. O. & Odom, B. C. Features of Molecular Structure Beneficial for Optical Pumping. *arXiv*, doi:10.48550/arxiv.2208.14296 (2022).

5 Li, A. & Guo, H. A Full-Dimensional Global Potential Energy Surface of $H_3O^+(\tilde{a}^3A)$ for the $OH^+(\tilde{X}^3\Sigma^-)$ + $H_2(\tilde{X}^1\Sigma_g^+)$ → $H(^2S)$ + $H_2O^+(\tilde{X}^2B_1)$ Reaction. *J. Phys. Chem. A* **118**, 11168-11176, doi:10.1021/jp5100507 (2014).

6 Shi, W., Jia, T. & Li, A. Quasi-Classical Trajectory Analysis with Isometric Feature Mapping and Locally Linear Embedding: Deep Insights into the Multichannel Reaction on An $NH_3^+(^4A)$ Potential Energy Surface. *Phys. Chem. Chem. Phys.* **22**, 17460-17471, doi:10.1039/d0cp01941k (2020).

7 Simonyan, V. V., Diep, P. & Johnson, J. K. Molecular Simulation of Hydrogen Adsorption in Charged Single-walled Carbon Nanotubes. *J. Chem. Phys.* **111**, 9778-9783, doi:10.1063/1.480313 (1999).

8 Buckingham, A. D. in *Advances in Chemical Physics* 107-142 (John Wiley & Sons, Inc., 2007).

9 Breneman, C. M. & Wiberg, K. B. Determining Atom-Centered Monopoles from Molecular Electrostatic Potentials - The Need for High Sampling Density in Formamide Conformational-Analysis. *J. Comput. Chem.* **11**, 361-373, doi:10.1002/jcc.540110311 (1990).

10 Knizia, G., Adler, T. B. & Werner, H.-J. Simplified CCSD(T)-F12 Methods: Theory and Benchmarks. *J. Chem. Phys.* **130**, 054104 (2009).

11 Pritchard, B. P., Altarawy, D., Didier, B., Gibson, T. D. & Windus, T. L. New Basis Set Exchange: An Open, Up-to-Date Resource for the Molecular Sciences Community. *J. Chem. Inf. Model.* **59**, 4814-4820, doi:10.1021/acs.jcim.9b00725 (2019).

12 Feller, D. & Peterson, K. A. An expanded calibration study of the explicitly correlated CCSD(T)-F12b method using large basis set standard CCSD(T) atomization energies. *J. Chem. Phys.* **139**, 084110 (2013).

13 Werner, H. J., Knowles, P. J., Knizia, G., Manby, F. R. & Schütz, M. Molpro: A General-Purpose Quantum Chemistry Program Package. *WIREs Comput. Mol. Sci.* **2**, 242-253 (2012).

14 Jiang, B., Li, J. & Guo, H. Potential Energy Surfaces From High Fidelity Fitting of Ab Initio Points: The Permutation Invariant Polynomial-Neural Network Approach. *Int. Rev. Phys. Chem.* **35**, 479 (2016).

15 Raff, L. M., Komanduri, R., Hagan, M. & Bukkapatnam, S. T. S. *Neural Networks in Chemical Reaction Dynamics*. (Oxford University Press, 2012).

16 Zhou, Z.-H., Wu, J. & Tang, W. Ensembling Neural Networks: Many Could Be Better Than All. *Art. Intel.* **137**, 239-263, doi:10.1016/S0004-3702(02)00190-X (2002).
